# Meta-Analysis of Materials and Treatments Used in Ophthalmic Lenses: Implications for Lens Characteristics

**DOI:** 10.3390/ma17235949

**Published:** 2024-12-05

**Authors:** Clara Martinez-Perez, Ana Paula Oliveira

**Affiliations:** 1 Instituto Superior de Educação e Ciências de Lisboa (ISEC Lisboa), Alameda das Linhas de Torres, 179, 1750-142 Lisboa, Portugal; clara.perez@iseclisboa.pt; 2 Centro de Investigação, Desenvolvimento e Inovação em Turismo (CiTUR)—Polo Estoril, Avenida Condes de Barcelona, n.º 808, 2769-510 Estoril, Portugal

**Keywords:** ophthalmic lenses, optical materials, lens coatings, lens characteristics, meta-analysis

## Abstract

Purpose: This meta-analysis evaluates the evolution, applications, and recent advancements in the materials and treatments used in ophthalmic lenses, focusing on their effectiveness, comfort, and safety, while also considering sustainability as a key factor. Methods: The study was registered with PROSPERO and conducted following the PRISMA and AMSTAR-2 guidelines. A systematic review was performed across databases such as PubMed, Web of Science, and Scopus, without time or language restrictions. Observational studies analyzing optical materials and ophthalmic lens treatments were included, and a random-effects model was applied due to the high heterogeneity observed among the studies. Results: Nine studies were included that evaluated different materials, such as CR-39^®^, PMMA with nanoparticles, and high-refractive-index polymers, with variability in refractive index (1.49 to 1.90) and light transmission (84.15% to 99%). Treatments like anti-reflective and abrasion-resistant coatings improved optical quality and durability. However, significant heterogeneity and publication bias were identified, limiting the generalizability of the results. Conclusions: The materials and treatments evaluated play a crucial role in the optical quality, durability, and comfort of lenses. Although advancements in sustainable materials show promise for reducing environmental impact, challenges remain in terms of cost and production. It is recommended to establish more stringent standards and promote research to improve the consistency and performance of ophthalmic lenses while ensuring sustainability.

## 1. Introduction

An estimated 2.2 billion people worldwide experience some level of vision impairment, affecting their ability to perform vision-dependent activities essential for daily living [1]. Approximately 80% of these cases are considered preventable or treatable with interventions like prescription spectacle lenses, contact lenses, or surgical procedures [2]. Spectacle lenses, or ophthalmic lenses, play a critical role in addressing refractive errors and restoring clear vision for individuals with conditions such as myopia, hyperopia, astigmatism, and presbyopia [3].

Ophthalmic lenses are primarily made from glass and various types of plastic. Originally, lenses were made exclusively of glass until the introduction of polymeric, or plastic, materials [3]. After World War I, advances in polymer technology resulted in new forms of plastics, with the most significant to the optical industry being the invention of polymerized methyl methacrylate (poly(methyl 2-methylpropenoate) or poly(methyl methacrylate), PMMA) in the 1930s [3,4]. Having good optical properties [5,6], it was used in the manufacture of spectacle lenses until the 1940s when an acrylic resin made of allyl diglycol carbonate (polyallyldiglycol carbonate, CR-39^®^) was developed and commercialized by PPG Industries [3,5,7,8]. Although CR-39^®^ is often used in the manufacture of ophthalmic lenses, newer materials like polycarbonate and Trivex^®^ have fewer crosslinks, and deliver a unique impact resistance. However, CR-39 lenses generally offer superior optical clarity, while polycarbonate may introduce slight optical distortion.

Plastic lenses have become highly popular, representing about 90% of the ophthalmic lens market [5]. Their widespread use is due to several advantages over traditional glass lenses, including superior impact resistance, lighter weight, and a thinner profile [3,8]. These benefits make polymer-based lenses the preferred choice for many eyewear applications. Nowadays, plastic ophthalmic lenses comprise polymers, or long-chain molecules that have many interconnecting branches or crosslinks, providing enhanced flexibility and resilience to impacts [3]. High-refractive-index plastic lenses are typically made of polyurethane resins [9], like poly(thio)urethanes (e.g., registered tradenames like Asahi-Lite^®^ and Tokai-UHI^®^), resulting in a lower density, light-weight lens material [5]. The use of nanoparticles in polymer nanocomposites can generate different properties in the final material, especially when used in hybrid systems in which synergistic effects can be produced. This innovation is achieved through the incorporation of nanoparticles with different methods [4,10] or the radical polymerization of different monomers [6,11].

In response to the demand for eco-friendly materials, new sustainable options have entered the ophthalmic lens market. One such innovation is the MR-174™, a bio-based high-refractive-index lens material that utilizes environmentally sustainable production methods [11]. Additionally, research by Pagliaro et al. [12] identifies several biopolymers with the potential for optical applications, including poly(limonene carbonate), silk fibroin, chitosan, gelatin, nanocellulose, alginate, polylactic acid, and polyhydroxyalkanoates. LTL, an Italian company specializing in ophthalmic and solar lenses, has introduced its “green lens” line, which incorporates plant-based materials to reduce plastic waste and carbon dioxide (CO_2_) emissions at a competitive price point [13]. These lenses include O-Green lenses, with up to 46% plant-derived carbon; M-Green lenses, with 90%; and P-Green lenses, with 10% [13].

To further enhance the durability and functionality of ophthalmic lenses, various treatments can be applied. These treatments can improve the lens’s resistance to scratches, impact resistance, reduce glare, and UV protection, or enhance other properties [3].

Anti-reflective coating (AR coating) reduces glare and reflections from light sources, which is particularly beneficial for night driving, working on computer screens, or any situation with significant overhead lighting. By minimizing reflections, these coatings improve visual clarity and cosmetic appearance, making the eyes more visible through the lenses. AR coatings are particularly important for high-index lenses, which tend to reflect more light. Studies have shown that AR coatings with a hard and hydrophobic surface have a wide range of applications, from enhancing visual comfort in optical devices to serving as antireflective covers on solar cells, with the added benefit of modest self-cleaning properties [14].

While no lens is completely scratch-proof, scratch-resistant coatings help protect plastic and other lens materials from everyday abrasions. These coatings are crucial for maintaining the optical clarity of lenses over time, especially for children’s eyewear, which is more likely to endure rough handling. Many lens materials, including polycarbonate and Trivex, naturally provide UV protection. For materials without inherent UV-blocking properties, a UV coating can be applied to filter out harmful UVA and UVB rays. Photochromic treatment adds convenience by combining prescription lenses with sun protection, reducing the need for a separate pair of sunglasses. However, they may not darken sufficiently in cars due to UV-blocking windshields. Lenses treated with anti-fog coatings prevent condensation on the lens surface in humid conditions or during temperature changes. This treatment is useful for those who frequently move between different environments or wear face masks. With the increase in screen time, blue light filtering coatings have become popular for reducing eye strain from digital devices. These coatings can help reduce glare and enhance contrast, potentially improving comfort for users who spend extended hours in front of screens.

This meta-analysis aims to evaluate the evolution, applications, and recent advancements in materials and treatments used for corrective eyewear, specifically focusing on ophthalmic lenses and coatings. The objective is to provide a comprehensive synthesis of the available data to understand how these innovations enhance vision correction, comfort, and safety, while also considering sustainability as a key factor in the development of future technologies.

## 2. Materials and Methods

### 2.1. Eligibility Criteria

**Figure 1:**
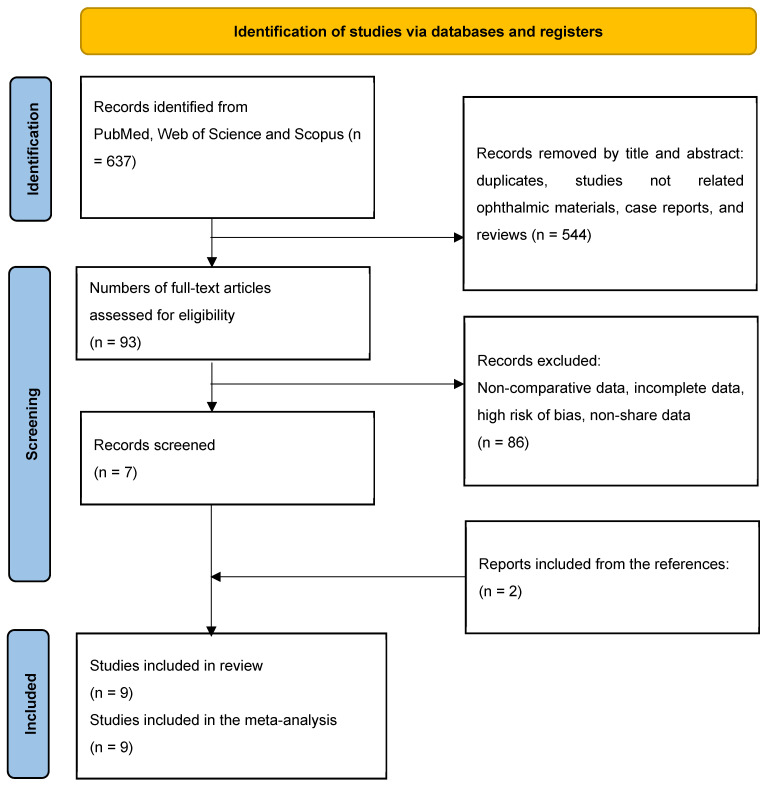
Flow diagram for study selection (following PRISMA guidelines for systematic reviews and meta-analyses).

This meta-analysis was registered in the International Prospective Register of Systematic Reviews (PROSPERO) under the registration number CRD42024602492 and conducted in accordance with the Preferred Reporting Items for Systematic Reviews and Meta-Analyses (PRISMA) guidelines [15] and adhering to the standards of A Measurement Tool to Assess Systematic Reviews 2 (AMSTAR-2) [16] (Figure 1). The research question was formulated using the PICOS strategy, focusing on the observational studies that evaluated ophthalmic lenses made from various optical materials and coatings, including PMMA, CR-39^®^, and nanocomposite materials. The intervention under investigation involved ophthalmic lenses composed of different monomers and treated with a range of optical coatings.

Comparators included lenses without special coatings or those with standard coating treatments, allowing a direct comparison of the technical properties across different treatments. The main outcomes of interest included light transmittance, refractive index, hardness, abrasion resistance, reflectance, and thickness of applied layers, all aimed at evaluating the materials’ and coatings’ effectiveness and durability under various conditions. Only the observational studies that quantitatively measured the optical materials’ properties under controlled laboratory conditions were included in the analysis.

### 2.2. Information Sources

An extensive literature review was carried out across multiple databases, including PubMed, Web of Science, and Scopus, using a systematic and rigorous search methodology. No restrictions were placed on time frame or language to ensure a comprehensive coverage of the relevant studies. Additionally, the reference lists of the initially selected studies were thoroughly reviewed to identify any further studies that may have been overlooked during the initial search phase.

### 2.3. Search Methods for Study Identification

The search strategy employed the following terms across all the trial records and databases: (Eco-friendly OR Sustainable OR Biodegradable OR Recycled OR Microplastic* OR Polymer* OR Plastic* OR Biomaterial*) AND (eyeglass OR “ophthalmic lens*”) (see Supplementary Materials S1). Two reviewers independently assessed the studies for eligibility, reaching a consensus on the final selection of studies to include.

### 2.4. Data Extraction and Data Items

Two reviewers independently reviewed the extracted data from the studies. The key information collected included the basic characteristics of each article, such as the study name, duration, geographic region, study design, sample size, and the types of materials used in the lenses. The primary variables compared across the studies were the refractive index and light transmittance of the materials and optical treatments.

### 2.5. Risk of Bias

**Figure 2:**
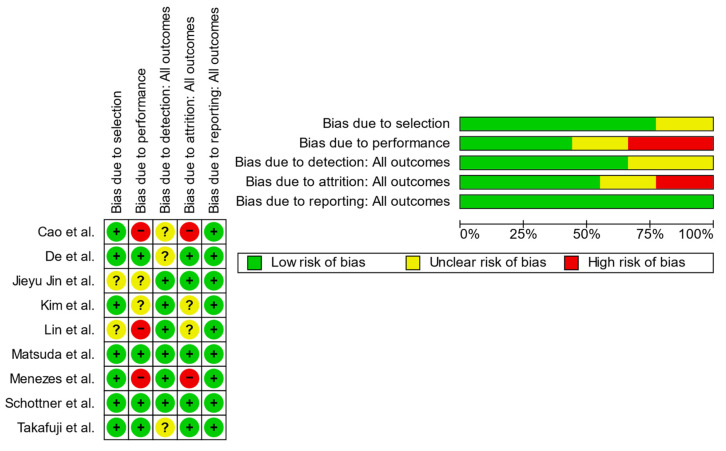
Risk of bias assessment (green = low risk; red = high risk; yellow = unknown) [4,5,6,8,10,14,17,18,19].

Two reviewers independently evaluated the methodological quality and risk of bias of the included studies using the Cochrane Collaboration’s Risk of Bias tool with the Review Manager 5.4 software. This tool systematically examines five key domains related to potential bias: material selection, reproducibility and consistency, objective assessment, completeness of data, and quality of reporting. Each domain was rated as having a low, high, or unclear risk of bias based on pre-defined criteria. The outcomes of the risk assessment are illustrated in Figure 2, with detailed justifications for each rating provided in Supplementary Materials S2.

### 2.6. Results Assessment

Mean differences (MDs) and 95% confidence intervals (CIs) were calculated for continuous variables measured on the same scale. For variables assessed on different scales, standardized mean differences (SMDs) were computed to facilitate comparison. Heterogeneity among the studies was evaluated using the I^2^ statistic, where values below 25% indicate low heterogeneity, values between 25% and 50% suggest moderate heterogeneity, and values above 50% indicate high heterogeneity. Due to the presence of significant heterogeneity, a random effects model (RE Model) was employed instead of a fixed-effects model. All the analyses were conducted using the JASP statistical software 0.19.1, which provides an open-source and user-friendly platform for advanced statistical analysis, ensuring the transparency and reproducibility of the results.

### 2.7. Publication Bias

A funnel plot analysis was conducted using the JASP software to evaluate potential publication bias. The asymmetry observed in the funnel plot may indicate publication bias, which can arise from the non-publication of smaller studies that report null or inconclusive results.

### 2.8. Additional Analyses

Subgroup analyses were conducted to investigate variations in outcomes based on several factors, including refractive index and transmittance. Additionally, a sensitivity analysis was performed by sequentially excluding the most heavily weighted studies within each subgroup. This approach aimed to verify the robustness of the results and assess how the exclusion of individual studies affected the overall conclusions.

## 3. Results

### 3.1. Study Selection

The initial search identified 637 articles (Figure 1). After removing duplicates, 54 studies were excluded, which included those not focused on ophthalmic lens materials or treatments, as well as case reports and review articles identified through title and abstract screening. This left 93 articles for further evaluation.

Upon reviewing the full texts, 86 studies were excluded for failing to meet the inclusion criteria, lacking comparable variables, or presenting incomplete data. Additionally, studies with a high risk of bias were excluded, as assessed using the Cochrane Collaboration’s Risk of Bias Tool. This tool evaluates five key domains: material selection, reproducibility and consistency, objective assessment, completeness of data, and quality of reporting. Studies were classified as having a high risk of bias if they exhibited issues such as inadequate methodological descriptions, incomplete or inconsistent data, or unclear outcome reporting. Furthermore, studies published in journals with limited peer-review processes or unclear editorial standards were also excluded. Ultimately, seven studies were included in the analysis. Additionally, two more studies were identified from the references of the included articles, resulting in a total of nine studies incorporated into the meta-analysis [3,4,5,6,7,8,9,10,11].

### 3.2. Study Characteristics

Table 1 provides a summary of the characteristics of the experimental studies included in this review, highlighting the key results reported in each study. Additionally, it includes the values for properties common to all the articles, such as refractive index and light transmittance. A total of nine studies were analyzed, each focusing on various types of lens materials and coatings. All the studies employed experimental designs, indicating a controlled methodology for evaluating the optical and mechanical properties of the materials.

The lens materials assessed included CR-39^®^, polymers with refractive index of 1.60 and 1.67, as well as PMMA enhanced with nanoparticles and other innovative compounds. The coatings varied from low-index layers to combinations of multiple materials, such as magnesium fluoride (MgF₂), indium tin oxide (ITO), and silicon dioxide (SiO₂), aimed at enhancing the durability and functionality of the lenses.

Key findings reveal that light transmission varied from 84.15% to 99% (Table 1), highlighting the effectiveness of the different treatments applied to the materials. Additionally, the refractive index of the materials ranged from 1.49 to 1.90 (Table 1), indicating a diversity in their composition and optical characteristics. 

### 3.3. Outcomes

#### 3.3.1. Different Types of Polymers

**Figure 3:**
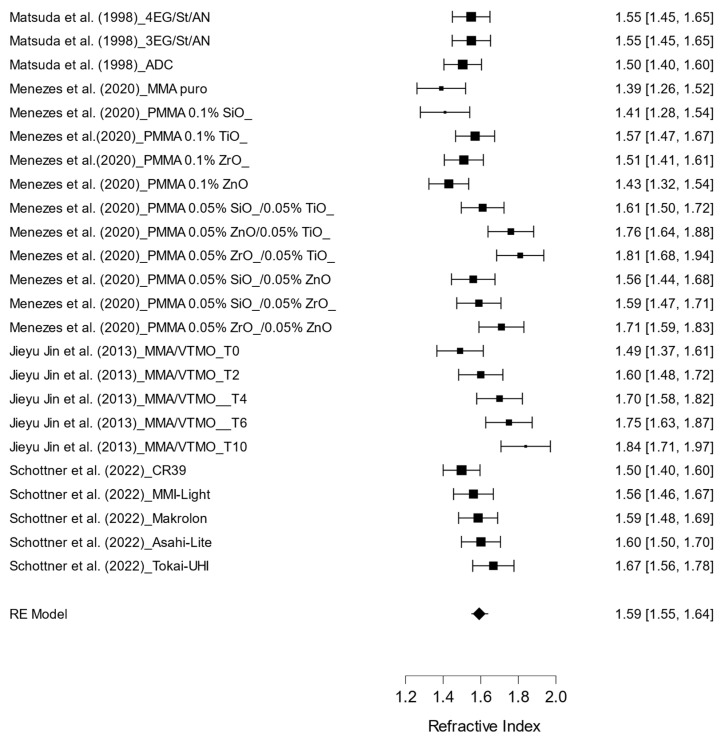
Effect of different types of polymers on the refractive index of ophthalmic lenses [5,6,8,10].

Figure 3 presents the pooled results of the studies examining the refractive index of various ophthalmic lens materials and their coatings. The evaluation included multiple materials, such as PMMA, CR-39^®^, methyl methacrylate (MMA), and hybrid polymers infused with various nanoparticles, all exhibiting refractive index ranging from 1.50 to 1.84. The overall mean refractive index was found to be 1.59 (95% CI: 1.55 to 1.64), indicating notable variability among the materials.

The high heterogeneity observed (I^2^ = 75.342%) among the studies suggests that differences in material formulation, nanoparticle concentration, and the coatings applied significantly influence the refractive index. Generally, materials with a higher concentration of components such as titanium dioxide (TiO₂) and zirconia Oxide (ZrO₂) tend to exhibit a greater refractive index.

**Figure 4:**
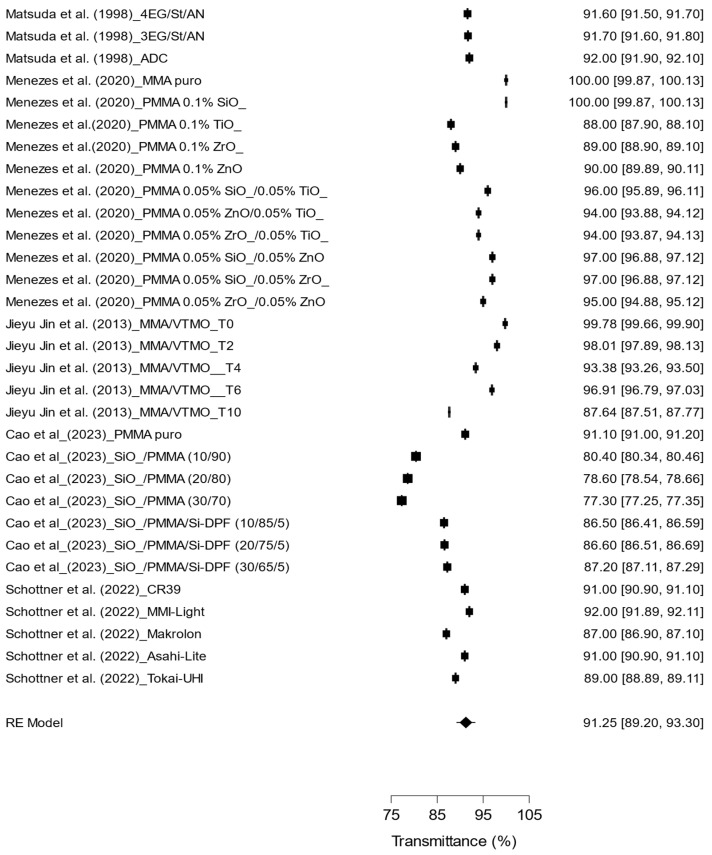
Effect of different types of polymers on light transmittance in ophthalmic lenses [4,5,6,8,10].

Figure 4 illustrates the pooled results of the studies examining light transmittance (%) across various ophthalmic lens materials. The evaluation encompassed a diverse range of combinations, including PMMA, CR-39^®^, MMA, and hybrid polymers incorporating nanoparticles. The findings indicate considerable variability in light transmittance among the different materials, with an overall mean of 91.25% (95% CI: 89.20 to 93.30). This suggests that, overall, these materials facilitate effective light transmission.

The heterogeneity among the studies was extremely high (I^2^ = 99.993%), indicating substantial variability in the materials analyzed. This pronounced heterogeneity can be attributed to differences in the characteristics of the materials and the methodologies employed in the studies.

Generally, materials with a greater number of combinations, particularly those containing multiple nanoparticles, tend to exhibit a reduction in light transmittance. This underscores the importance of finding a balance between light transmission and other optical properties, such as impact resistance and reflection reduction. The findings suggest that both the type of material used and the coating strategy directly influence the effectiveness of the lens in terms of light transmission, which is critical for its application in ophthalmic optics.

##### Meta-Regression Analysis

**Figure 5:**
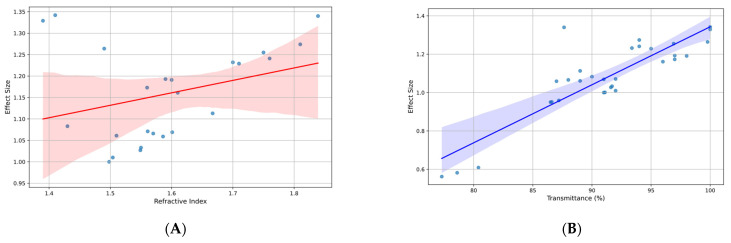
(**A**) Effect size vs. refractive index in studies on polymers for ophthalmic lenses; (**B**) effect size vs. transmittance (%) in studies on polymers for ophthalmic lenses.

The meta-regression analysis evaluated the impact of refractive index and transmittance on effect size (Figure 5). Both predictors showed a significant relationship with effect size. The refractive index had a coefficient of 0.4324 (95% CI: 0.158 to 0.707; *p* = 0.004), while transmittance showed a coefficient of 0.0196 (95% CI: 0.011 to 0.028; *p* < 0.001). The model explained 58.7% of the variability in effect size (R^2^ = 0.587).

##### Sensitivity Analysis

To assess the robustness of the results, the studies with the highest standard errors (90th percentile) were excluded. After this exclusion, the model fit improved, with an adjusted R^2^ of 78.1%. The coefficient for refractive index increased to 0.5001 (95% CI: 0.289 to 0.711; *p* < 0.001), and for transmittance, it was 0.0159 (95% CI: 0.010 to 0.022; *p* < 0.001). This analysis confirmed that the results are consistent and not influenced by studies with high imprecision.

##### Subgroup Analysis

The materials were grouped into two categories based on the refractive index: <1.5 and ≥1.5. The materials with a refractive index ≥ 1.5 showed a higher average effect size (1.342) compared to those with a refractive index < 1.5 (1.118). Therefore, the materials with a higher refractive index are associated with better optical performance, likely due to their ability to minimize chromatic aberrations and allow for thinner lenses.

#### 3.3.2. Different Types of Treatments

**Figure 6:**
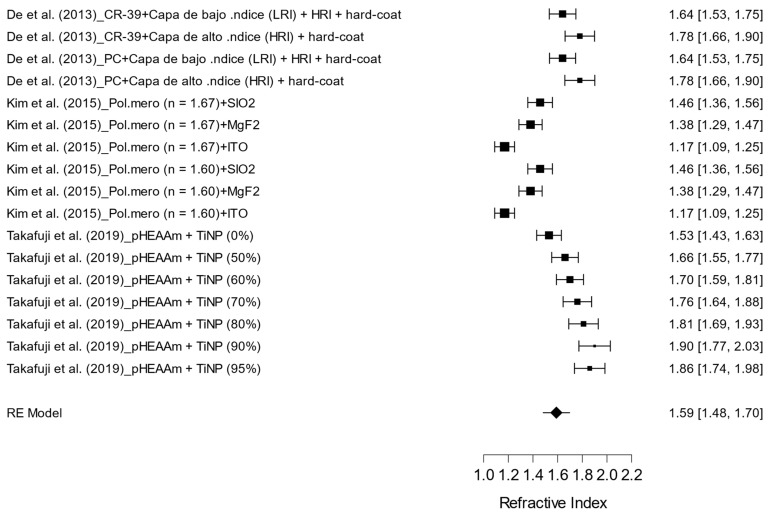
Effect of different types of ophthalmic lens treatments on the refractive index [14,18,19].

The pooled results concerning the refractive index of various treatments applied to optical lenses are presented in Figure 6. A total of 18 treatments were analyzed, encompassing combinations of polymeric materials and specific coatings, including both high- and low-index layers, hybrid materials, and polymer combinations with varying proportions of titanium dioxide nanoparticles (TiNPs).

The effect size analysis revealed variations in refractive index estimates, ranging from 1.17 to 1.90. Overall, the findings indicate a pooled mean refractive index of 1.59 (95% CI: 1.48 to 1.70), suggesting significant enhancements in refractive index due to the application of these different treatments. The observed heterogeneity was high (I^2^ = 94.59%), indicating considerable variability among the included studies.

Notably, specific treatments such as poly (N-hydroxyethyl acrylamide) (pHEAAm) combined with 90% and 95% TiNPs demonstrated higher refractive index, underscoring the potential for significant improvements with these combinations. The results from the random effects model (RE Model) indicate that despite the heterogeneity, there is a consistent effect of refractive index enhancement across the analyzed treatments.

**Figure 7:**
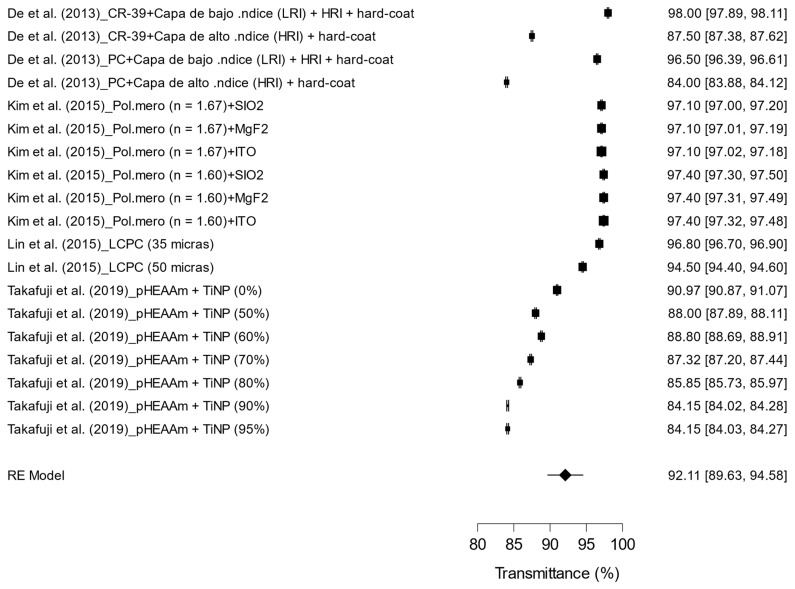
Effect of treatments on light transmittance in ophthalmic lenses [14,17,18,19].

The pooled results for light transmittance across the different lens treatments are illustrated in Figure 7. The studies included in the analysis evaluated a variety of materials and coatings, ranging from CR-39^®^ with high- or low-index layers to polymers combined with agents such as SiO₂, MgF₂, and TiNP. The effect sizes varied from 84.00% to 98.00%, highlighting the differences in the effectiveness of the treatments for enhancing light transmittance. The random effects model (RE Model) yielded a mean transmittance value of 92.11% with a 95% confidence interval of [89.63, 94.58]. Significant heterogeneity was observed (I^2^ = 99.99%), indicating substantial variability among the studies, likely due to the diverse materials and coating techniques employed. These findings suggest that certain treatments, particularly those featuring high-index coatings, demonstrate higher transmittance levels, which positively influence the optical quality of the lenses.

##### Meta-Regression

**Figure 8:**
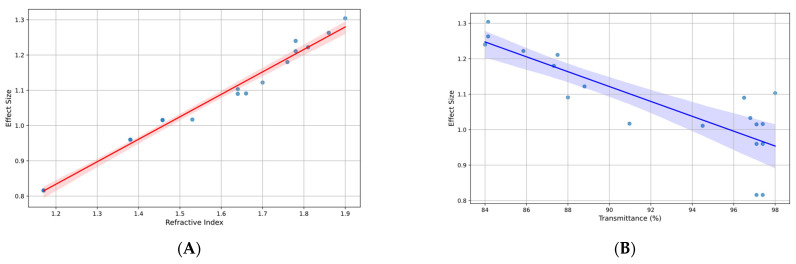
(**A**) Effect size vs. refractive index in studies on treatments for ophthalmic lenses; (**B**) effect size vs. transmittance (%) in studies on treatments for ophthalmic lenses.

A meta-regression analysis was conducted to evaluate the influence of refractive index and transmittance on effect size (Figure 8). The results showed that the refractive index had a positive and significant impact (coefficient = 0.6201; 95% CI: 0.527 to 0.713; *p* < 0.001), while transmittance was not statistically significant (coefficient = −0.0008; 95% CI: −0.005 to 0.003; *p* = 0.648). The model explained 98.1% of the variability in effect size (R^2^ = 0.981), highlighting the relevance of the refractive index as a key factor in the optical performance of the evaluated materials.

##### Sensitivity Analysis

To assess the robustness of the results, a sensitivity analysis was performed by excluding the studies with the highest standard errors (90th percentile). After the exclusion, the model retained a high explanatory power (adjusted R^2^ = 97.3%), and the refractive index remained a significant predictor (coefficient = 0.6119; 95% CI: 0.516 to 0.708; *p* < 0.001). Transmittance continued to show no statistical significance (*p* = 0.832). This analysis confirmed the consistency of the results regardless of the influence of the studies with lower precision.

##### Subgroup Analysis

The results were analyzed by dividing the data into two subgroups based on refractive index: materials with indices < 1.5 and materials with indices ≥ 1.5. The materials with refractive indices ≥ 1.5 exhibited a higher average effect size (1.211) compared to the materials with indices < 1.5 (1.118). This suggests that the materials with higher refractive indices are associated with better optical performance, likely due to their ability to reduce chromatic aberrations and enable thinner lenses.

#### 3.3.3. Publication Bias

**Figure 9:**
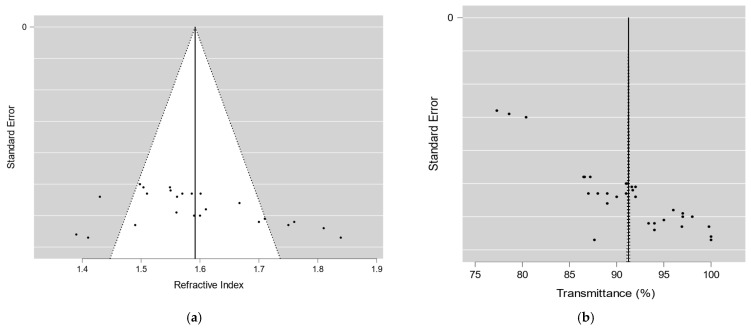
Funnel plots to assess publication bias in studies on polymers used in ophthalmic lenses: (**a**) bias assessment for refractive index; (**b**) bias assessment for optical transmittance.

The funnel plots in Figure 9 depict the potential publication bias in the studies examining the optical performance of polymers used in lenses. For the refractive index, the plotted points exhibit some asymmetry around the effect line, which may suggest the presence of publication bias. However, this asymmetry could also be attributed to methodological differences across studies or variations in the properties of the polymers analyzed.

The funnel plot for transmittance reveals greater dispersion, particularly in the extreme values (Figure 9), indicating that factors such as study quality or sample size may have affected the likelihood of certain studies being published. This potential bias should be considered when assessing the robustness of the meta-analysis results. The observed asymmetry suggests that studies with more favorable or significant findings might be overrepresented, potentially skewing the overall conclusions about polymer performance.

**Figure 10:**
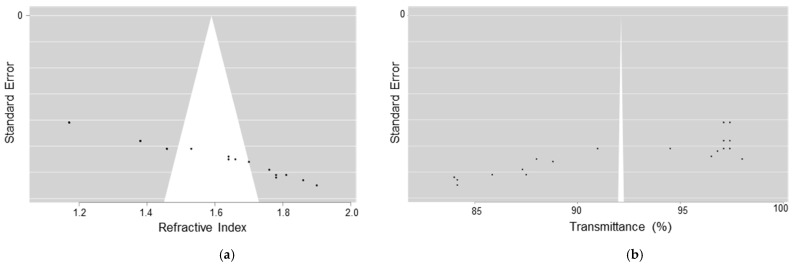
Funnel plot illustrating publication bias in the studies of lens treatments, with values falling within an acceptable range: (**a**) bias assessment for the refractive index across various lens treatments; (**b**) bias assessment for transmittance across different lens treatments.

The funnel plots in Figure 10 indicate the potential presence of publication bias in the studies on lens treatments. For the refractive index, the plot displays an asymmetric distribution around the effect line, which may suggest publication bias, although this asymmetry could also result from study heterogeneity. In contrast, the funnel plot for transmittance reveals a wider spread at the extremes, further suggesting the possibility of bias.

The asymmetry observed in both plots indicates that bias may be present in the published studies, implying that research with significant results or larger effect sizes might have had a higher chance of being published. This potential bias should be considered when interpreting the findings of the meta-analysis, as it could impact the reliability of the conclusions drawn.

## 4. Discussion

In this study, a detailed evaluation of the materials, treatments, and sustainability approaches applied to ophthalmic lenses is presented, providing a comparative and updated overview of their effectiveness and potential impact on the optical industry.

The materials evaluated in ophthalmic lenses, including CR-39^®^, PMMA with nanoparticles, and high-refractive-index polymers, exhibit a wide variability in refractive index (1.49 to 1.90) and light transmission (84.15% to 99%). This range highlights the diversity in the compositions and optical performance of the materials. According to Pillay et al. [3], advancements in the industry have led to a significant shift from glass to polymers like CR-39^®^ and polycarbonate due to their light weight and durability. However, in this study, advanced polymers with nanoparticles offer an advantage in terms of strength and visual clarity. Chandrinos et al. [20] also analyze how high-refractive-index polymers, including the use of epoxy resins and sulfur-containing components, allow for a higher refractive index and reduced chromatic aberration. Our results reflect this progress, with nanoparticle-enhanced materials such as TiO₂ and ZrO₂ demonstrating improved refractive indices without significantly increasing lens weight. However, the heterogeneity observed in light transmission and visual clarity suggests that further optimization of composition and the standardization of the manufacturing process are needed to ensure consistent performance.

Polymers with a refractive index greater than two are a cutting-edge area of materials science with transformative potential for advanced optical applications, particularly in scenarios demanding compact, high-performance lenses. Achieving such high refractive indices typically requires organic–inorganic hybrid materials, such as nanocomposites, which involve embedding high-refractive-index nanoparticles into polymer matrices [21]. However, their development faces significant challenges, including non-uniform film formation, increased optical loss due to Rayleigh scattering, and the complexity of the preparation process, which can hinder scalability and practicality [21]. Addressing these limitations is crucial to unlocking their potential for ultra-thin lenses, precision optical devices, and emerging technologies like augmented and virtual reality systems.

The materials evaluated in ophthalmic lenses, including CR-39^®^, PMMA with nanoparticles, and high-refractive-index polymers, demonstrate a broad variability in refractive index (1.49 to 1.90) and light transmission (84.15% to 99%). This range highlights the diversity in the compositions and optical performance of the materials. According to Bohling et al.’s [22] study on abrasion-resistant optical coatings, the incorporation of multilayer coatings of SiO₂ and TiO₂ on ophthalmic lenses significantly improves light transmission, achieving transmittance levels above 98%, which is consistent with our findings. These coatings not only enhance visual clarity but also offer greater durability against wear.

Wu et al. [23] also address the importance of abrasion-resistant coatings for protecting the integrity of lenses, especially in environments where wear is a critical issue. Our findings reflect the effectiveness of these coatings in reducing wear effects on polymer lenses, suggesting that combining high-refractive-index materials with abrasion-resistant coatings could provide an optimal solution for improving both durability and visual clarity under prolonged use conditions.

The study by Rampersad and Carlson [24] evaluated the spectral transmission of commercial lenses designed to filter high-energy visible (HEV) light, commonly known as blue light. Their findings indicated that while these lenses effectively block blue light, they also introduce significant variations in the transmission of other wavelengths, potentially impacting color perception and overall visual clarity. Similarly, our results indicate that nanoparticle-enhanced materials, such as TiO₂ and ZrO₂, improve the refractive index of lenses without a significant increase in weight. However, we also noted heterogeneity in light transmission and visual clarity, suggesting the need to optimize composition and standardize the manufacturing process to ensure consistent performance. The variability in the effectiveness of blue light filters underscores the importance of the thorough evaluation of lens optical properties to ensure adequate eye protection without compromising visual quality.

Regarding the treatments applied to the lenses, anti-reflective coatings and blue light filters demonstrated high effectiveness. Our analysis indicates that anti-reflective coatings, especially those composed of MgF₂ and SiO₂ layers, significantly reduced reflections and improved light transmission, with performance reaching up to 99%. This aligns with the review from Raut et al. [22], which highlights the implementation of multilayer coatings to minimize reflectance through physical vapor deposition techniques. Thus, while advanced materials, such as high-index polymers with nanoparticles, are making strides in optical quality, challenges remain regarding performance consistency. The lack of standardization in the manufacturing process and in the formulations of these materials impacts the uniformity of their optical properties, which is critical in ophthalmic applications that require a high degree of precision. The industry would benefit from establishing stricter standards and fostering research to develop polymers with stable, reproducible characteristics that reliably meet user needs across diverse settings.

Sustainability is becoming an increasingly important focus in ophthalmic lens design. Pagliaro et al. [12] explore the potential of biopolymers, such as nanocellulose and poly(limonene carbonate), which are renewable materials offering promising optical properties like high transparency and efficient light guidance. Although biopolymers are not yet widely used in the ophthalmic lens industry, they hold substantial potential for reducing environmental impact, particularly if their production can be made cost-effective. Chandrinos et al. [20] further highlight materials like MR-174™, a plant-derived, high-refractive-index polymer, as a sustainable alternative with optical performance comparable to conventional materials. However, the high costs and production challenges associated with these materials remain significant barriers. Another critical aspect of sustainability in the ophthalmic sector is the issue of lens recycling. Recycling is complicated by the diversity of lens materials, including substrates, inorganic coatings, and tints, which often make lenses incompatible with standard recycling methods (Oliveira et al., 2024). Polymer lenses, for example, can be categorized as thermoplastic (such as polyurethane and polycarbonate) or thermo-hardened (such as CR-39^®^). Recycling is feasible only for thermoplastic lenses, and even then, it depends on the specific surface treatments they have undergone [25]. Moreover, the optical industry currently exhibits limited sustainability practices. Oliveira et al. [25] found that 40.8% of the surveyed Portuguese Optical Centers do not engage in any form of recycling for optical materials, reflecting a significant gap in environmental stewardship within the sector. For the ophthalmic lens industry to transition toward greater sustainability, it is essential to invest in continued research and innovation. This includes developing biodegradable and eco-friendly materials that meet the industry’s stringent standards for quality and durability, as well as implementing more effective recycling practices to reduce waste.

This study has several limitations that should be taken into account when interpreting the findings. First, there was considerable heterogeneity across the analyzed studies, particularly regarding light transmission and refractive index, making generalization challenging due to the variations in material compositions and manufacturing methods. Additionally, the risk of publication bias may have led to an overrepresentation of studies with favorable results, potentially affecting the reliability of the conclusions. The lack of production standardization also impacts the consistency of optical properties, which is crucial for ophthalmic applications. Moreover, high costs and industrial barriers currently limit the widespread adoption of sustainable materials. Another limitation is that the analysis primarily focused on two common properties—refractive index and light transmittance—since these were consistently reported across all the studies. Other relevant properties, such as abrasion resistance, reflectance, or thermal stability, were not considered in the meta-analysis due to inconsistencies in measurement methods or the absence of data in several studies. Lastly, the absence of clinical studies restricts the practical applicability of these findings in real-world use. Despite these limitations, this study provides a comprehensive overview that can guide future research aimed at improving the quality and sustainability of ophthalmic lenses.

## 5. Conclusions

The materials and treatments used in ophthalmic lenses play a vital role in shaping their performance, durability, and comfort, directly impacting the wearer’s visual experience. The choice of lens material should consider the wearer’s lifestyle, prescription strength, and personal preferences. Advanced treatments, such as anti-reflective, scratch-resistant, and UV-blocking coatings, further enhance lenses by improving protection and optimizing visual performance. Thoughtfully combining materials and treatments allows eyewear to meet both functional and aesthetic demands, providing the wearer with superior optical quality and comfort.

In recent decades, the field of vision correction has undergone transformative advancements, from new materials to innovative manufacturing techniques, paving the way for a future where emerging technologies will redefine vision correction even further. As we look toward the future, it is essential to develop technologies and materials that not only advance optical performance but also uphold sustainability, ensuring a positive impact on both users and the planet’s ecological balance.

## Figures and Tables

**Table 1:** Baseline characteristics of studies on optical materials and coating.

Article	Year	Type of Study	Type of Lens Material and/or Coating *	Article Key Results	Refractive Index	Light Transmittance (%)
Lin et al. [17]	2015	Experimental	CR-39^®^ + LCPCF (35 and 50 μm)	97% transmittance, low roughness	1.498	99%
De et al. [14]	2013	Experimental	CR-39^®^ + LRI + HRI	2.9% reflectance (400–800 nm)	1.6	98%
Menezes et al. [8]	2020	Experimental	PMMA with nanoparticles	Improve hardness and transparency	1.53	92%
Matsuda et al. [6]	1998	Experimental	Polymer: tetraethylene glycol dimethacrylate or triethylene glycol dimethacrylat + styrene or a-methylstyrene + acrylonitrile	High impact resistance, good heat resistance	1.508	91%
Kim et al. [18]	2015	Experimental	Polymer (n = 1.67 and n = 1.60) + MgF₂ + ITO + SiO_2_	High energy visible light blocking	1.67	98%
Schottner et al. [5]	2003	Experimental	CR-39^®^	High hardness, improved durability	1.49	90%
Cao et al. [4]	2023	Experimental	PMMA	Improve hardness, reduction in reflection	1.65	91.1%
Jieyu Jin et al. [10]	2013	Experimental	MMA/VTMO (racio 7:1)	Improve thermal stability	1.7	93.38%
Takafuji et al. [19]	2019	Experimental	pHEAAm + TiNP	High refractive index	1.9	84.15%

* HRI—solution prepared using tetraethyl orthosilicate (TEOS), (3-glycidoxypropyl)trimethoxysilane (GLYMO), titanium tetraisopropoxide (TTIP), acetylacetone (acac), n-butanol, water, HNO_3_, and aluminum acetylacetonate (Al(acac)3); LCPCF—liquid crystal and polymer composite film; LRI—solution prepared using TEOS, GLYMO, n-butanol, water, methanol, nitric acid (HNO_3_), and Al(acac)3; MMA—Methyl methacrylate; PMMA—Poly(methyl methacrylate); VTMO—Vinyltrimethoxysilane.

## Data Availability

The original contributions presented in this study are included in the article/Supplementary Materials. Further inquiries can be directed to the corresponding author.

## Supplementary Materials

The following supporting information can be downloaded at https://www.mdpi.com/article/10.3390/ma17235949/s1, Supplementary Materials S1: PubMed search strategy; Supplementary Materials S2: Risk of bias judgement.
