# Supplementary material for: Meta-Analysis of Materials and Treatments Used in Ophthalmic Lenses: Implications for Lens Characteristics
Source: Materials (Basel). 2024 Dec 5;17(23):5949. doi: 10.3390/ma17235949 (PMC11643459; doi:10.3390/ma17235949)
Supplement: Supplementary file 1 [file materials-17-05949-s001.zip › Supplementary material s1.pdf]

Supplementary File 1. PubMed search strategy.

Search: **(Eco-friendly OR Sustainable OR Biodegradable OR Recycled OR Microplastic* OR Polymer* OR Plastic* OR Biomaterial*) AND (eyeglass OR "ophthalmic lens*")**
("Eco-friendly"[All Fields] OR ("sustain"[All Fields] OR "sustainability"[All Fields] OR "sustainable"[All Fields] OR "sustainably"[All Fields] OR "sustained"[All Fields] OR "sustaining"[All Fields] OR "sustainment"[All Fields] OR "sustains"[All Fields]) OR ("biodegradabilities"[All Fields] OR "biodegradability"[All Fields] OR "biodegradable"[All Fields] OR "biodegradables"[All Fields] OR "biodegradations"[All Fields] OR "biodegradative"[All Fields] OR "biodegrade"[All Fields] OR "biodegradeable"[All Fields] OR "biodegraded"[All Fields] OR "biodegrader"[All Fields] OR "biodegraders"[All Fields] OR "biodegrades"[All Fields] OR "biodegrading"[All Fields] OR "metabolism"[MeSH Subheading] OR "metabolism"[All Fields] OR "biodegradation"[All Fields] OR "biodegradation, environmental"[MeSH Terms] OR ("biodegradation"[All Fields] AND "environmental"[All Fields]) OR "environmental biodegradation"[All Fields]) OR ("recyclability"[All Fields] OR "recyclable"[All Fields] OR "recyclables"[All Fields] OR "recyclate"[All Fields] OR "recyclates"[All Fields] OR "recycler"[All Fields] OR "recyclers"[All Fields] OR "recycles"[All Fields] OR "recycling"[MeSH Terms] OR "recycling"[All Fields] OR "recycle"[All Fields] OR "recycled"[All Fields] OR "recyclings"[All Fields]) OR "microplastic*"[All Fields] OR "polymer*"[All Fields] OR "plastic*"[All Fields] OR "biomaterial*"[All Fields]) AND ("eyeglasses"[MeSH Terms] OR "eyeglasses"[All Fields] OR "eyeglass"[All Fields] OR "ophthalmic lens*"[All Fields])
Translations
Sustainable: "sustain"[All Fields] OR "sustainability"[All Fields] OR "sustainable"[All Fields] OR "sustainably"[All Fields] OR "sustained"[All Fields] OR "sustaining"[All Fields] OR "sustainment"[All Fields] OR "sustains"[All Fields]
Biodegradable: "biodegradabilities"[All Fields] OR "biodegradability"[All Fields] OR "biodegradable"[All Fields] OR "biodegradables"[All Fields] OR "biodegradations"[All Fields] OR "biodegradative"[All Fields] OR "biodegrade"[All Fields] OR "biodegradeable"[All Fields] OR "biodegraded"[All Fields] OR "biodegrader"[All Fields] OR "biodegraders"[All Fields] OR "biodegrades"[All Fields] OR "biodegrading"[All Fields] OR "metabolism"[Subheading] OR "metabolism"[All Fields] OR "biodegradation"[All Fields] OR "biodegradation, environmental"[MeSH Terms] OR ("biodegradation"[All Fields] AND "environmental"[All Fields]) OR "environmental biodegradation"[All Fields]
Recycled: "recyclability"[All Fields] OR "recyclable"[All Fields] OR "recyclables"[All Fields] OR "recyclate"[All Fields] OR "recyclates"[All Fields] OR "recycler"[All Fields] OR "recyclers"[All Fields] OR "recycles"[All Fields] OR "recycling"[MeSH Terms] OR "recycling"[All Fields] OR "recycle"[All Fields] OR "recycled"[All Fields] OR "recyclings"[All Fields]
eyeglass: "eyeglasses"[MeSH Terms] OR "eyeglasses"[All Fields] OR "eyeglass"[All Fields]
