# Supplementary material for: Meta-Analysis of Materials and Treatments Used in Ophthalmic Lenses: Implications for Lens Characteristics
Source: Materials (Basel). 2024 Dec 5;17(23):5949. doi: 10.3390/ma17235949 (PMC11643459; doi:10.3390/ma17235949)
Supplement: Supplementary file 1 [file materials-17-05949-s001.zip › Supplementary material s2.pdf]

Supplementary File 2. Risk of bias judgement.

1. Menezes et al. (2020)

Criterion	Evaluation	Justification
**Bias due to selection**	Low	The selection of materials (PMMA with various nanoparticles) is clearly justified and aligned with the study's goals.
**Bias due to performance**	High	There is a lack of detailed description about reproducibility and consistency across tests, leading to performance concerns.
**Bias due to detection**	Low	The optical and mechanical properties were measured using objective and standardized methods, minimizing detection bias.
**Bias due to attrition**	High	No information on missing data or how incomplete data were managed, which leads to attrition bias.
**Bias due to reporting**	Low	The study reports key outcomes transparently and clearly, reducing the risk of bias from selective reporting.

2. Matsuda et al. (1998)

Criterion	Evaluation	Justification
**Bias due to selection**	Low	Materials are clearly selected and relevant for the scope of the study.
**Bias due to performance**	Low	Detailed descriptions and consistent findings indicate that the study is reproducible and has low performance bias.
**Bias due to detection**	Low	Objective measures, such as refractive index and hardness, were assessed using validated methods, minimizing detection bias.
**Bias due to attrition**	Low	No issues with incomplete data were reported, and the study presents a full dataset.
**Bias due to reporting**	Low	Results were reported completely and transparently, with no signs of selective reporting.

3. Jieyu Jin et al. (2013)

Criterion	Evaluation	Justification
**Bias due to selection**	Unclear	The selection process for materials is not entirely clear, leading to uncertainty in this domain.
**Bias due to performance**	Unclear	There is insufficient detail about how consistent the performance of the materials was across tests.
**Bias due to detection**	Low	Objective measurements were made using standardized methods, reducing detection bias.
**Bias due to attrition**	Low	No evidence of missing data was reported, indicating a complete dataset.
**Bias due to reporting**	Low	The study reports outcomes clearly and does not appear to have selective reporting.

4. Cao et al. (2023)

Criterion	Evaluation	Justification
**Bias due to selection**	Low	The study provides clear justification for the selection of PMMA and additives, leading to low selection bias.
**Bias due to performance**	High	Performance details, particularly regarding reproducibility, are not sufficiently detailed, raising concerns about bias.

**Bias due to detection**	Unclear	Some measurement methods are not fully detailed, leaving room for potential detection bias.
**Bias due to attrition**	High	No information is provided on how missing or incomplete data were handled, raising attrition bias concerns.
**Bias due to reporting**	Low	The study reports key outcomes transparently, reducing the risk of selective reporting bias.

5. Schottner et al. (2022)

**Criterion**	**Evaluation**	**Justification**
**Bias due to selection**	Low	The selection of materials is clearly justified and relevant to the study's objectives.
**Bias due to performance**	Low	The study is consistent in its methods, and reproducibility is evident across the tests.
**Bias due to detection**	Low	Objective measurements using standardized techniques were used, minimizing detection bias.
**Bias due to attrition**	Low	There is no evidence of missing data, and all results are reported completely.
**Bias due to reporting**	Low	The study provides transparent and thorough reporting, reducing the risk of reporting bias.

6. De et al. (2013)

**Criterion**	**Evaluation**	**Justification**
**Bias due to selection**	Low	The materials were selected appropriately for the study's goals, reducing the risk of selection bias.
**Bias due to performance**	Low	The study provides adequate details about reproducibility and consistency across the tests.
**Bias due to detection**	Unclear	Some aspects of the objective assessment are not fully explained, leading to potential detection bias.
**Bias due to attrition**	Low	No missing data is reported, leading to a low risk of bias due to attrition.
**Bias due to reporting**	Low	The study reports key outcomes fully and transparently, reducing the risk of selective reporting bias.

7. Kim et al. (2015)

**Criterion**	**Evaluation**	**Justification**
**Bias due to selection**	Low	The study justifies the selection of materials clearly and aligns with the research objectives.
**Bias due to performance**	Unclear	Insufficient details are provided on the reproducibility of the results, leading to uncertainty in performance bias.
**Bias due to detection**	Low	The study uses objective measures and standardized methods, reducing detection bias.
**Bias due to attrition**	Unclear	There is limited information on how missing data were handled, leaving room for attrition bias.
**Bias due to reporting**	Low	The study reports its outcomes clearly and transparently, with no evidence of selective reporting.

8. Lin et al. (2015)

**Criterion**	**Evaluation**	**Justification**
**Bias due to selection**	Unclear	The rationale for the selection of materials is not fully described, leading to uncertainty about selection bias.
**Bias due to performance**	High	Performance consistency is not adequately addressed, raising concerns about reproducibility.
**Bias due to detection**	Low	Objective measures were used, with standardized methods reducing detection bias.

**Bias due to attrition**	Unclear	No information is provided on how incomplete or missing data were handled.
**Bias due to reporting**	Low	Reporting is clear and transparent, with no evidence of selective reporting.

9. Takafuji et al. (2019)

**Criterion**	**Evaluation**	**Justification**
**Bias due to selection**	Low	The study provides clear justification for the materials used, reducing the risk of selection bias.
**Bias due to performance**	Low	The study provides consistent and reproducible results, with clear descriptions of methods.
**Bias due to detection**	Unclear	While objective methods were used, insufficient detail was provided to fully assess detection bias.
**Bias due to attrition**	Low	No evidence of missing data is reported, leading
